# Quantumness Measures for a System of Two Qubits Interacting with a Field in the Presence of the Time-Dependent Interaction and Kerr Medium

**DOI:** 10.3390/e23050635

**Published:** 2021-05-19

**Authors:** Sayed Abdel-Khalek, Kamal Berrada, Eied M. Khalil, Abdel-Shafy F. Obada, Esraa Reda, Hichem Eleuch

**Affiliations:** 1 Department of Mathematics and Statistics, College of Science, Taif University, P.O. Box 11099, Taif 21944, Saudi Arabia; sabotalb@tu.edu.sa (S.A.-K.); eiedkhalil@tu.edu.sa (E.M.K.); 2 Department of Physics, College of Science, Imam Mohammad Ibn Saud Islamic University (IMSIU), Riyadh 11432, Saudi Arabia; 3 The Abdus Salam International Centre for Theoretical Physics, Strada Costiera 11, 34151 Miramare-Trieste, Italy; 4 Department of Mathematics, Faculty of Science, Al-Azhar University, Cairo 13759, Egypt; asobada@yahoo.com; 5 Department of Mathematics, Faculty of Education, Ain Shams University, Cairo 11566, Egypt; esraareda226@yahoo.com; 6 Department of Applied Physics and Astronomy, University of Sharjah, Sharjah 27272, United Arab Emirates; heleuch@sharjah.ac.ae; 7 Department of Applied Sciences and Mathematics, College of Arts and Sciences, Abu Dhabi University, Abu Dhabi 59911, United Arab Emirates; 8 Institute for Quantum Science and Engineering, Texas A & M University, College Station, TX 77843, USA

**Keywords:** atom-field systems, Tavis-Cummings model, time-dependent interaction, Kerr medium, entropy, negativity, Mandel’s parameter, 03.67.-a, 03.65.Yz, 03.65.Ud

## Abstract

In this work, we introduce the standard Tavis-Cummings model to describe two-qubit system interacting with a single-mode field associated to power-law (PL) potentials. We explore the effect of the time-dependent interaction and the Kerr-like medium. We solve the Schrödinger equation to obtain the density operator that allows us to investigate the dynamical behaviour of some quantumness measures, such as von Neumann entropy, negativity and Mandel’s parameter. We provide how these entanglement measures depend on the system parameters, which paves the way towards better control of entanglement generation in two-qubit systems. We find that the enhancement and preservation of the atoms-field entanglement and atom-atom entanglement can be achieved by a proper choice of the initial parameters of the field in the absence and presence of the time-dependent interaction and Kerr medium. We examine the photons distribution of the field and determine the situations for which the field exhibits super-poissonian, poissonian or sub-poissonian distribution.

## 1. Introduction

One of the strongest features of quantum mechanics is the quantum entanglement, which Schrödinger first introduced in 1935 [1]. The phenomenon of quantum entanglement can be obtained with a special superposition method for several wave functions that cannot be written as a product of subsystem wave functions. Therefore, the subject of quantum entanglement is considered one of the most basic topics in quantum information science, such as quantum computation and communication [2], simultaneous transmission [3], dense coding [4] and coding [5]. Several schemes have been proposed for generating entanglement of photons by bimodal superposition of coherent states [6], squeezed states [7], and superposition of states [8] and nonlinear coherent states [9]. On the other hand, there are practical attempts to generate superpositions of two-mode entangled states [10] and GHZ-type and W-type entangled coherent states [11]. Whereas for two photons moving in multiple directions, this is a case of entanglement that contains linear momentum and degrees of polarization freedom [12]. In recent years, it has been realized that orbital angular momentum is another degree of freedom of the radiation field which can be used to generate entanglement [13].

Recently, non-classical light plays a significant role in distinguishing between classical physics and quantum physics. So far, the non-classical state of lights and atoms has become the key center in different tasks of quantum information and optics [14]. The characteristics of non-classical lights are similar to the classical analysis of electromagnetic waves and are described by quantum mechanics theory. Recently, some works have significantly demonstrated the microscopic impact of the nonclassical properties of photons on the improvement of the spectral resolution [15], field imaging [16], wavelength measurement with antibunching [17,18], coherent effects [19], propagation [20]. This shows that the non-classical nature of the light is a broad topic, and scientists are working hard to better explore the inward truth of the quantum world.

Last decades, coherent states played a vital role in various branches of physics [21,22], and they are introduced as the eigenvectors of the lowering operators of the harmonic oscillators [23]. These quantum states have physical features similar to classical electromagnetic fields. In this case, the classical trajectory is utilized to characterize the center of the coherent state wave packet associated with the harmonic oscillator potential. There exist coherent states that describe nonlinear electromagnetic fields with nonclassical properties including antibunching and squeezing [24,25]. Taking into account nonclassical quantum effects, the classical and non-classical limits of the quantized field are specified by the Glauber coherent state.

The PL potentials have attracted a lot of attention and gained more and more insights on various physics topics [26,27,28]. PL potentials can introduce a large set of realistic potentials, such as triangular, harmonic and infinite potential. A comparative study of these potentials has been demonstrated through the exponent parameter. In this context, like harmonic oscillator coherent states (CSs), the CSs used for PL potentials can be helpful in quantum information and optics. In fact, it seems that apart from their theoretical ability, the form of PL potential CSs may also have practical significance in helping to better understanding the behavior and characteristics of the quantum system considered in the framework of the JC and TC models. Using the PL potentials, we present a valuable and relevant new study of entanglement measures for a system of two qubits interacting with a field. We extend the usual TC model by considering two qubits when they simultaneously interact with a radiation field in PL potentials and taking into account the effect of the time-varying coupling and Kerr-like term. We solve the Schrödinger equation to obtain the density operator that allows us to investigate the dynamical behavior of the quantumness measures such as von Neumann entropy, negativity and Mandel’s parameter. We show how the quantumness measures for the proposed scheme can be affected by the main parameters of the physical model, and we compare the results to the case of fields for different values of the exponent parameter of the PL potential. The proposed model can be useful to understand some quantum-mechanical phenomena of nonlinear optics.

The paper is organized as follows. In Section 2, we describe the PL potentials and its CSs. In Section 3, we introduce the physical model and system dynamics. In Section 4, we describe the quantumness measures and the obtained results. A brief conclusion is given in the last section.

## 2. Power-Law Potentials

The general expression of a one-dimensional PL potential is introduced as

(1) $$V\left(x,k\right)=V_{o}{\left|\frac{x}{a}\right|}^{k},$$

where $V_{o}$ and *a* have the dimensions of energy and length, respectively. The parameter *k* is a positive real number that is called the power-law exponent. These PL potentials can be utilized to introduce a large class of quantum systems through a proper choice of the exponent *k*.

The Hamiltonian associated with PL potentials is defined by

(2) $$\hat{H}=\frac{{\hat{p}}^{2}}{2m}+\hat{V}\left(x,p\right),$$

where its corresponding eigenvalue equations are given by

(3) $$\hat{H}\left(k\right)|n\rangle =E_{n,k}|n\rangle ,\;\;\;\;\;n\geq 0.$$

The Fock states $|n\rangle$ are the eigenstates and $E_{n,k}$ are its corresponding eigenenergies.

Substituting Equation (2) into Equation (3), we obtain

(4) $${\hat{p}}^{2}|n\rangle =\left[2m(E_{n,k}-\hat{V})\right]|n\rangle ,$$

where

(5) $$p\left(x\right)=\sqrt{2m(E_{n}-V)}.$$

The eigen-energy spectrum $E_{n,k}$ can be obtained by using the Wentzel-Kramers-Brillouin (WKB) approximation [29,30,31], such that

(6) $$\int_{-x_{o}}^{+x_{o}}p\left(x\right)dx=\left(n+\frac{g}{4}\right)\pi \hbar ,$$

where $\pm x_{o}$ are the classical turning points. Here, *g* is the Maslov index, which accounts for the boundary effects at the classical turning points.

In the case of E=V(x), we have

(7) $$\pm x_{o}=\pm a{\left(\frac{E}{V_{o}}\right)}^{\frac{1}{k}}.$$

Using Equations (1) and (5), the Equation (6) can be written as

(8) $$2\int_{0}^{x_{o}}\sqrt{2m\left(E_{n}-V_{o}{\left(\frac{x}{a}\right)}^{k}\right)}dx=\left(n+\frac{g}{4}\right)\pi \hbar .$$

This integral can be solved using the substitution, $u={\left(\frac{x}{a}\right)}^{k}$ with $dx=\frac{a}{k}u^{\frac{1}{k}-1}du$, and we have

(9) $$\frac{2a\sqrt{2m}}{k}\int_{0}^{\frac{E}{V_{o}}}\sqrt{\left(E_{n}-V_{o}u\right)}u^{\frac{1}{k}-1}du=\left(n+\frac{g}{4}\right)\pi \hbar ,$$

where

(10) $$\int_{0}^{\frac{E}{V_{o}}}\sqrt{\left(E_{n}-V_{o}u\right)}u^{\frac{1}{k}-1}du=\frac{1}{V_{o}^{\frac{1}{k}}}\frac{\Gamma \left(\frac{1}{k}\right)\Gamma \left(\frac{2}{3}\right)}{\Gamma (\frac{1}{k}+\frac{2}{3})}E_{n}^{\frac{1}{k}+\frac{1}{2}}.$$

Therefore, the eigenenergy spectrum is given by

(11) $$\begin{array}{ccc} E_{n,k} & = & {\left[\left(n+\frac{g}{4}\right)\pi \hbar \frac{kV_{o}^{\frac{1}{k}}}{2a\sqrt{2m}}\frac{\Gamma (\frac{1}{k}+\frac{2}{3})}{\Gamma \left(\frac{1}{k}\right)\Gamma \left(\frac{2}{3}\right)}\right]}^{\frac{2k}{k+2}},\\ & = & \omega \left(k\right){\left(n+\frac{g}{4}\right)}^{\frac{2k}{k+2}}, \end{array}$$

where $\omega \left(k\right)={\left[\frac{\pi \hbar }{2a\sqrt{2m}}V_{o}^{\frac{1}{k}}\frac{\Gamma (\frac{1}{k}+\frac{2}{3})}{\Gamma \left(\frac{1}{k}+1\right)\Gamma \left(\frac{2}{3}\right)}\right]}^{\frac{2k}{k+2}}$ is the effective frequency.

The parameter *k* determines the type of the potentials. To gain insight into the structure of the energy spectrum given by Equation (11), we consider the energy difference between levels

(12) $$\begin{array}{ccc} ▵E_{n}^{\left(k\right)} & = & E_{n}^{\left(k\right)}-E_{n-1}^{\left(k\right)}\\ & \propto & {\left(n+\frac{g}{4}\right)}^{\frac{k-2}{k+2}}. \end{array}$$

Equation (12) shows that for k=2, $▵E_{n}^{\left(k\right)}$ is independent on *n*, so the energy spectrum is equally spaced. For the exponent k≠2, the level spacing varies with *n*. For k>2, the energy difference increases with *n* (tightly binding potentials), while for k<2, the energy between adjacent levels decreases with *n* (loosely binding potentials).

The CSs associated with PL potentials are defined by [26,27,28]

(13) $$|Z,k\rangle =N_{Z,k}\sum_{n=0}^{\infty }\frac{Z^{n}}{\sqrt{G(n,k)}}|n\rangle ,$$

where

(14) $$G\left(n,k\right)=\prod_{i=1}^{n}\left[{\left(i+\frac{g}{4}\right)}^{\frac{2k}{k+2}}-{\left(\frac{g}{4}\right)}^{\frac{2k}{k+2}}\right],\;\;\;\;G\left(0,k\right)=1,$$

and the normalization function $N_{Z,k}$ is

(15) $$N_{Z,k}={\left[\sum_{n=0}^{\infty }\frac{{\left|Z\right|}^{2n}}{G(n,k)}\right]}^{-\frac{1}{2}}.$$

For k=2, the PL potential reduces to harmonic oscillator potential and Equation (13) becomes the standard coherent states for the harmonic oscillator [32]. In the k→∞ limit, the PL potential becomes the infinite square-well potential [33]. For K=1, the PL potential reduces to the triangular-well potential [34].

## 3. System Hamiltonian and Dynamics

The proposed model considers a system of two atoms (qubits) that interact with a quantized field initially prepared in CSs associated with PL potentials in the presence of a Kerr-like medium. Many previous studies dealt with time dependence in different ways, including [35]. The time dependence of this model was derived without the nonlinearity of termes in [36,37]. Under the rotating-wave approximation, The system Hamiltonian can be written as

(16) $$\hat{H}=\nu {\hat{A}}^{✝}\hat{A}+\frac{1}{2}\sum_{j=1}^{2}\omega_{j}{\hat{\sigma }}_{z}^{\left(j\right)}+\chi \hat{n}\left(\hat{n}-1\right)+\lambda \cos\left(pt\right)\sum_{j=1}^{2}\left(\hat{A}{\hat{\sigma }}_{+}^{\left(j\right)}+{\hat{A}}^{+}{\hat{\sigma }}_{-}^{\left(j\right)}\right),$$

where the time-dependent (independent) coupling case is occurred when p≠0 (p=0 ). χ describes the effect of the Kerr-like medium, $\sigma_{z}^{\left(j\right)}$, ${\hat{\sigma }}_{\pm }^{\left(j\right)}$ are the standard qubit transition operators for the *j*th qubit, $\hat{A}$ and ${\hat{A}}^{+}$ are the annihilation and creation operators of the generalized Heisenberg algebra [26], respectively. The qubit transition frequency is $\omega_{j}$, ν is the field frequency and λ is a coupling constant between each atom and the field.

We assume that the two atoms are initially in the upper state $|u_{AB}\left(0\right)\rangle =|e_{1},e_{2}\rangle$ and the field in the state [38]

(17) $$|u_{F}\left(0\right)\rangle =\frac{1}{\sqrt{1+h^{2}+2h\langle Z,k|-Z,k\rangle }}\left[\sum_{n=0}^{\infty }\beta_{n}\left\{1+h{\left(-1\right)}^{n}\right\}|n\rangle \right],$$

where

$$\beta_{n}=\frac{Z^{n}}{\sqrt{G(n,k)}}.$$

The state (17) is corresponding to CSs of PL potentials for h=0 and a superposition (even) of CSs for h=1.

At subsequent times t>0, the state vector of the whole system is given by

(18) $$\begin{array}{ccc} |u(t)\rangle & = & \sum_{n=0}^{\infty }K_{1}\left(n,t\right)|e_{1},e_{2},n\rangle +K_{4}\left(n,t\right)|g_{1},g_{2},n+2\rangle \\ & & +\left\{K_{2}\left(n,t\right)|e_{1},g_{2}\rangle +K_{3}\left(n,t\right)|g_{1},e_{2}\rangle \right\}|n+1\rangle . \end{array}$$

The coefficients $K_{j}\left(n,t\right)$ can be obtained by set of ordinary differential equation resulting from the Schrödinger equation:

(19) $$i\frac{dK}{dt}=\Lambda K,$$

So

(20) $$K=\left(\begin{array}{c} K_{1}\\ K_{2}\\ K_{3}\\ K_{4} \end{array}\right)\;\mathrm{and}\;\Lambda =\left(\begin{array}{cccc} \chi n(n-1) & \lambda \sqrt{n+1} & \lambda \cos\left(pt\right)\sqrt{n+1} & 0\\ \lambda \cos\left(pt\right)\sqrt{n+1} & \chi n(n+1) & 0 & \lambda \cos\left(pt\right)\sqrt{n+2}\\ \lambda \cos\left(pt\right)\sqrt{n+1} & 0 & \chi n(n+1) & \lambda \cos\left(pt\right)\sqrt{n+2}\\ 0 & \lambda \cos\left(pt\right)\sqrt{n+2} & \lambda \cos\left(pt\right)\sqrt{n+2} & \chi (n^{2}+3n+2) \end{array}\right),\;$$

with the initial condition $K_{1}\left(n,0\right)=\beta_{n}$ and $K_{l}\left(n,0\right)=0$ with l=2,3,4.

The atomic density matrix can be obtained by evaluating the trace over the field basis

(21) $${\hat{\rho }}_{AB}\left(t\right)={\mathrm{Tr}}_{\mathrm{Field}}\left\{|u(t)\rangle \langle u(t)|\right\},$$

where the diagonal elements of the two atoms density matrix are given by

(22) $$\rho_{ll}=\sum_{n=0}^{\infty }{\left|K_{l}\left(n,t\right)\right|}^{2},\;\;\;l=1,2,3,4,$$

while the off-diaconal elements are satisfying $\rho_{ij}=\rho_{ji}^{*}$ and have the form

(23) $$\begin{array}{ccc} \rho_{12} & = & \sum_{n=1}^{\infty }K_{1}\left(n,t\right)K_{2}^{*}\left(n-1,t\right),\;\;\;\;\rho_{13}=\sum_{n=1}^{\infty }K_{1}\left(n,t\right)K_{3}^{*}\left(n-1,t\right),\; \end{array}$$

(24) $$\begin{array}{ccc} \rho_{14} & = & \sum_{n=2}^{\infty }K_{1}\left(n,t\right)K_{4}^{*}\left(n-2,t\right),\;\;\;\;\rho_{23}=\sum_{n=0}^{\infty }K_{2}\left(n,t\right)K_{3}^{*}\left(n,t\right), \end{array}$$

(25) $$\begin{array}{ccc} \rho_{24} & = & \sum_{n=1}^{\infty }K_{2}\left(n,t\right)K_{4}^{*}\left(n-1,t\right),\;\;\;\;\rho_{34}=\sum_{n=1}^{\infty }K_{3}\left(n,t\right)K_{4}^{*}\left(n-1,t\right). \end{array}$$

## 4. Quantum Quantifiers and Main Results

In this section, we use the derived density matrix of the two atoms (21) to discuss the atoms-field entanglement as well as atom-atom entanglement. Moreover, we consider Mandel’s parameter to examine the quantum statistics of the quantized field. We assume that the atoms have equal transition energies.

To quantify the entanglement of the atoms-field state, we use the von Neumann entropy that is given by

(26) $$S_{N}\left(t\right)=-\mathrm{Tr}\left[{\hat{\rho }}_{AB}\left(t\right)\ln{\hat{\rho }}_{AB}\left(t\right)\right].$$

**Figure 1:**
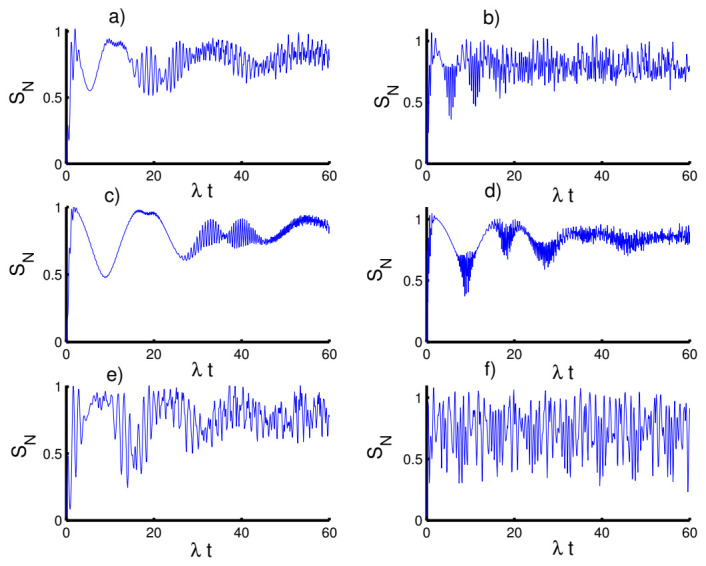
Time evolution of the atomic entropy $S_{N}\left(t\right)$ for the physical parameters with the values (Z, *p*, χ)=(3,0,0). (**a**,**c**,**e**) The field initially in the CSs for PL potentials (h=0) and (**b**,**d**,**f**) show the field initially in the superposition of CSs for PL potentials (h=1). (**a**,**b**) for harmonic well potential (k=2, g=2), (**c**,**d**) show the triangular well (k=1, g=3), and (**e**,**f**) show the infinite well (k→∞, g=4).

In Figure 1, the behavior of the function $S_{N}\left(t\right)$ is displayed with fixed parameters (Z, *p*, χ)=(3,0,0). In the case of the harmonic well potential (k=2) and a coherent state (h=0), a strong entanglement is generated between the field and the two atoms. Interestingly, the minimum values of entanglement are achieved in the middle of the collapse and revival period and the maximum values are achieved outside this period. On the other hand, the amount of the entanglement can be enhanced and the intensity of oscillations increases after setting the field in the even coherent state (h=1). For the triangular well (k=1) with a coherent state (h=0), the amount of the entanglement increases accompanied by a decrease in the intensity of the oscillations. When the field state starts from the even coherent state (h=1), the amount of the entanglement decreases with an increase in the amplitude of oscillations. For the infinite well (k→∞) with a coherent state (h=0), the entanglement decreases and the intensity of its oscillations increases. On the other hand, the entanglement is enhanced with a decrease in the amplitude of oscillations after the field starts from the even coherent state (h=1).

**Figure 2:**
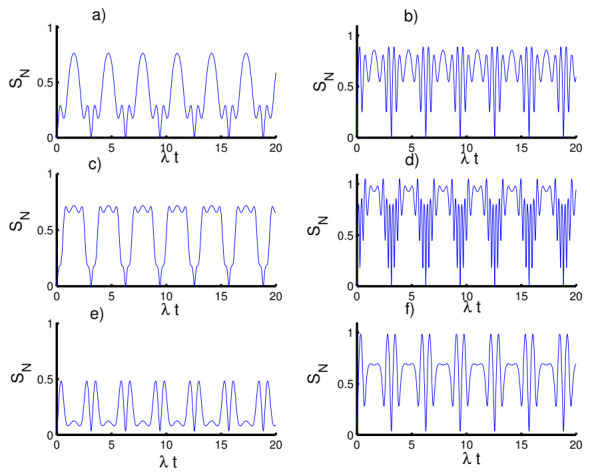
Effect of time-dependent coupling (p=1) on the evolution of the atomic entropy $S_{N}\left(t\right)$ for the physical parameters with the values (Z, χ)=(3,0). (**a**,**c**,**e**) The field initially in the CSs for PL potentials (h=0) and (**b**,**d**,**f**) show the field initially in the superposition of CSs for PL potentials (h=1). (**a**,**b**) Harmonic well potential (k=2, g=2), (**c**,**d**) triangular well (k=1, g=3), and (**e**,**f**) infinite well (k→∞, g=4).

In Figure 2, we show the effect of the time-dependent interaction on the function $S_{N}\left(t\right)$ considering the same previous conditions. In the first case (k=2,h=0), the entanglement decreases smoothly and it reaches periodically the minimum values for every time π and maximum values for every $\frac{(2n+1)\pi }{2}$ with (n=0,1,2,...). The entanglement significantly enhanced and the intensity of oscillations increases when the field state starts from the even coherent state (h=1). Concerning the second case (k=1,h=0), there is a further enhancement in the amount of the entanglement over most of the interaction period. After setting the field in the even coherent state, the values of the function $S_{N}\left(t\right)$ can be increased in some time intervals with more regular oscillations compared to the previous case. In the third case (k→∞, h=0), the amount of the entanglement decreases and it is completely improved when the field setting in the even coherent state.

**Figure 3:**
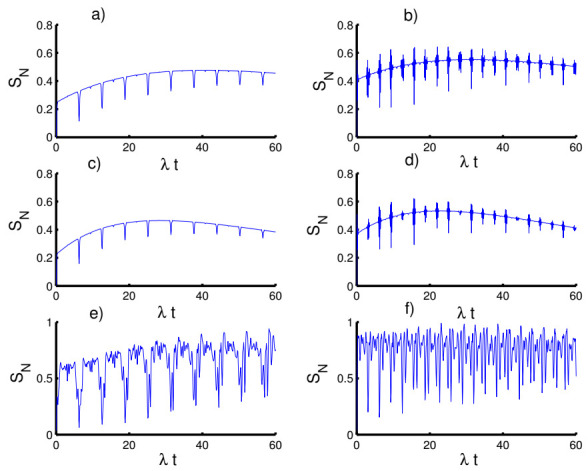
Effect of Kerr medium (χ=0.5λ) on the evolution of the atomic entropy $S_{N}\left(t\right)$ for the physical parameters with the values (Z, p)=(3,0). (**a**,**c**,**e**) The field initially in the CSs for PL potentials (h=0) and (**b**,**d**,**f**) show the field initially in the superposition of CSs for PL potentials (h=1). (**a**,**b**) Harmonic well potential (k=2, g=2), (**c**,**d**) the triangular well (k=1, g=3), and (**e**,**f**) the infinite well (k→∞, g=4).

In Figure 3, we explore the effect of the Kerr medium on the entanglement of the atoms-field state. In the case of k=2 with h=0, the presence of the Kerr medium results in a restraint of the entanglement and stabilizes the behavior of its quantifier, $S_{N}\left(t\right)$, during the evolution. On the other hand, the entanglement can be improved when the field starts from the even coherent state (h=1). Moreover, we can observe that the effect of the Kerr medium decreases with increasing time. For the second case, k=1 with h=0, the function $S_{N}\left(t\right)$ behaves in a similar way as the previous case. While the third case, k→∞ with h=0, shows that the effect of the Kerr medium is weak. Moreover, the entanglement increases gradually as the interaction time evolves. In the even coherent state, a strong entanglement appears and the effect of the Kerr medium disappears almost as observed in the Figure 3f.

In order to discuss the dynamics of the entanglement of the atom-atom state, we use the negativity as a measure and it is defined by [39]

(27) $$N\left(\rho_{AB}\right)=\frac{∥\rho_{AB}^{T_{A}}∥-1}{2},$$

in which $\rho_{AB}^{T_{A}}$ is the partial transpose of $\rho_{AB}$ with respect to the first atom. Negativity varies from $N(\rho_{AB})=0$ to 1 corresponding to an unentangled state and maximally entangled state (EPR states), respectively.

**Figure 4:**
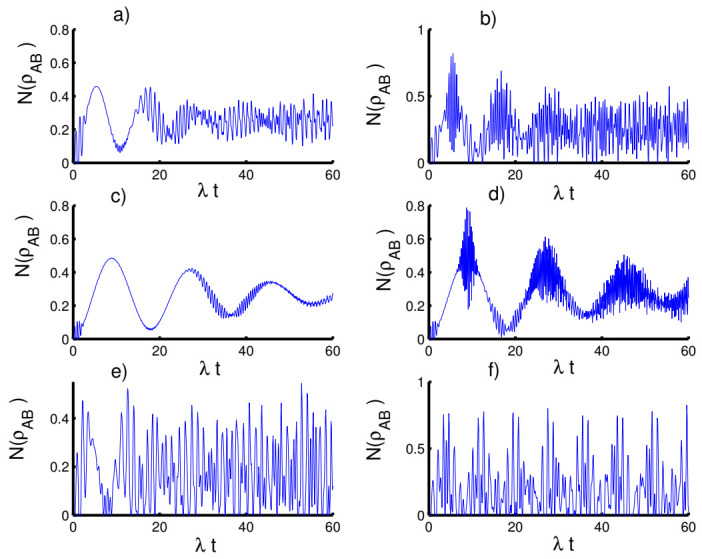
The evolution of the negativity $N(\rho_{AB})$ for the physical parameters with the values (Z, χ,p)=(3,0,0). (**a**,**c**,**e**) The field initially in the CSs for PL potentials (h=0) and (**b**,**d**,**f**) show the field initially in the superposition of CSs for PL potentials (h=1). (**a**,**b**) Harmonic well potential (k=2, g=2), (**c**,**d**) triangular well (k=1, g=3), and (**e**,**f**) infinite well (k→∞, g=4).

In Figure 4, we plot the time evolution of the negativity of the atom-atom state versus time with respect to the fixed parameter values. For the first case, k=2 with h=0, we can observe a monotonic relation appears between the atomic entropy and the negativity. Entanglement is generated during the interaction and the function $N\left(\rho_{AB}\right)$ fluctuates between 0 and 0.5. Interestingly, the entanglement reaches maximum values at mid-collapse time and also before and after revival time. For the even coherent state (h=1), the entanglement can be slightly enhanced at the beginning of the interaction and the phenomena of sudden death and sudden birth of entanglement appear. In the second case, k=1 with h=0, the amount of entanglement increases, the fluctuation intensity of the function $N\left(\rho_{AB}\right)$ increases and the death and sudden birth phenomena disappear. In the third case, k→∞ with h=0, random entanglement is generated with an increase in the amplitude of oscillations with the existence of phenomena of sudden death and sudden birth. When setting the field in the even coherent state, the amount of entanglement is clearly improved accompanied by an increase in the phenomena of sudden death and sudden birth of the entanglement.

**Figure 5:**
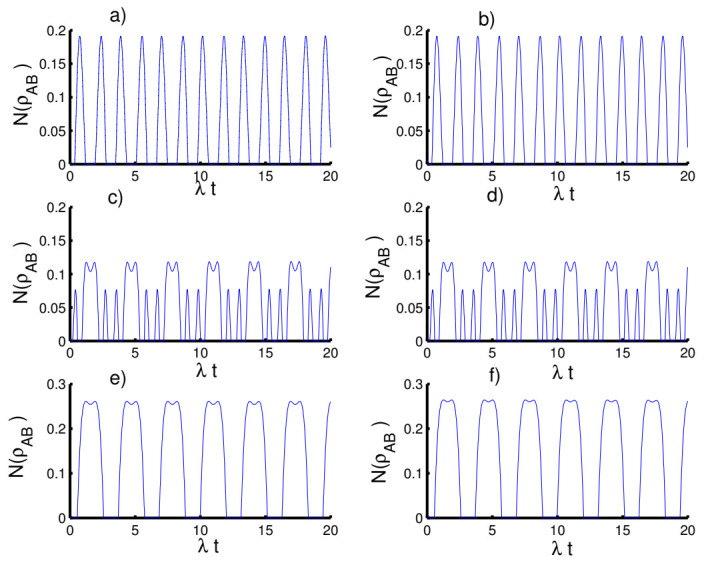
Effect of time-dependent coupling on the evolution of the negativity $N(\rho_{AB})$ for the physical parameters with the values (Z, χ)=(3,0). (**a**,**c**,**e**) The field initially in the CSs for PL potentials (h=0) and (**b**,**d**,**f**) show the field initially in the superposition of CSs for PL potentials (h=1). (**a**,**b**) Harmonic well potential (k=2, g=2), (**c**,**d**) triangular well (k=1, g=3), and (**e**,**f**) infinite well (k→∞, g=4).

In Figure 5, we display the influence of the time-dependent interaction on the entanglement of the atom-atom state. In general, the function $N\left(\rho_{AB}\right)$ exhibits a periodic behavior with sudden death and sudden birth phenomena of entanglement in the presence of the time-dependent interaction for various cases of the field state. Moreover, the previous chaotic oscillations become ordered with the reduction of the intensity of the oscillations.

**Figure 6:**
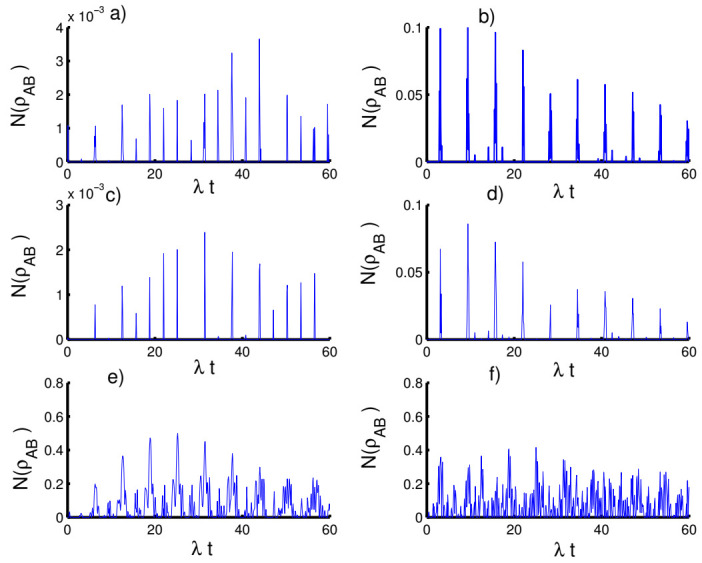
Effect of Kerr medium (χ=0.5λ) on the evolution of the negativity $N(\rho_{AB})$ for the parameters with the values (Z, p)=(3,0). (**a**,**c**,**e**) The field initially in the CSs for PL potentials (h=0) and (**b**,**d**,**f**) for the field initially in the superposition of CSs for PL potentials (h=1). (**a**,**b**) Harmonic well potential (k=2, g=2), (**c**,**d**) triangular well (k=1, g=3), and (**e**,**f**) infinite well (k→∞, g=4).

In Figure 6, we plot the results that show the effect of the Kerr medium on the entanglement of the atom-atom state. It is found that the presence of the Kerr medium causes disentanglement between the two atoms, especially when preparing the field in the coherent state (h=0) for the three kinds of potentials. On the other hand, the amount of the entanglement can be enhanced in the case of the even coherent state (h=1) accompanied by an increase in the periods of sudden death and sudden birth.

Let us now analyze the nonclassicality of the field state through the Mandel parameter that includes the statistical properties of the photons, specifically bunching and antibunching of the photons. The Mandel parameter is defined as [40]

(28) $$P_{M}=\frac{\langle {\left({\hat{A}}^{✝}\hat{A}\right)}^{2}\rangle }{\langle {\hat{A}}^{✝}\hat{A}\rangle }-\langle {\hat{A}}^{✝}\hat{A}\rangle -1.$$

Depending on the value of the parameter $P_{M}$, we can distinguish the photon statistics of the field with $P_{M}=0$ for the Poissonian distribution case, whereas $P_{M}>0$ and $P_{M}<0$ correspond to the super-Poissonian and sub-Poissonian cases, respectively.

**Figure 7:**
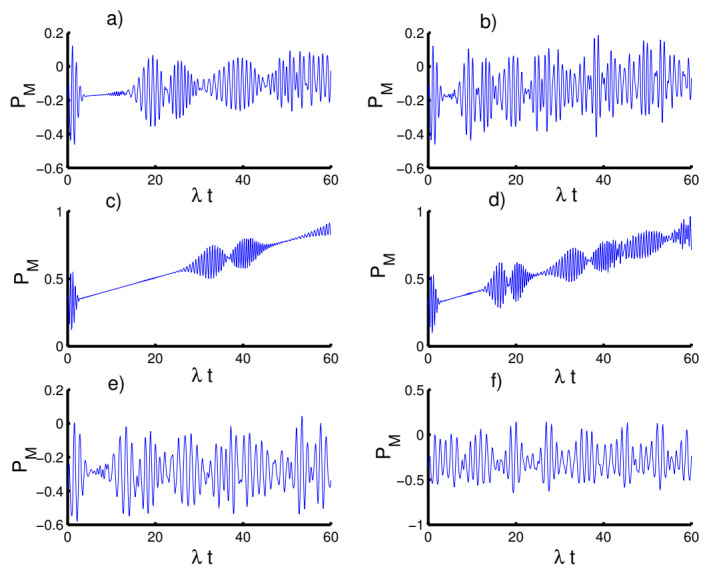
The evolution of the Mandel parameter $P_{M}$ for the physical parameters with the values (Z, χ,p)=(3,0,0). (**a**,**c**,**e**) The field initially in the CSs for PL potentials (h=0) and (**b**,**d**,**f**) show the field initially in the superposition of CSs for PL potentials (h=1). (**a**,**b**) Harmonic well potential (k=2, g=2), (**c**,**d**) triangular well (k=1, g=3), and (**e**,**f**) infinite well (k→∞, g=4).

In Figure 7, we display the time variation of Mandel’s parameter with respect to the different values of the physical parameters. In the absence of the time-dependent interaction and Kerr medium effects, the Mandel parameter exhibits the classical distribution of the photons for most of the time of the interaction. In the first case (k=2, h=0), the non-classical (sub-Poissonian) distribution achieves in small periods before and after the mid-revival period. The non-classical distribution gradually occurs with increasing time of interaction when preparing the field in the even coherent state (h=1). In the second case (k=1), the situation changes completely and the Mandel parameter takes positive values for both cases of the field state, h=0 and h=1, satisfying super-Poissonian distribution. The Mandel parameter again provides the classical distribution in the third case of k→∞ for fields with h=0 and h=1. On the other hand, the periods of the non-classical distribution are greatly reduced with an increase in the amplitude of the oscillations.

**Figure 8:**
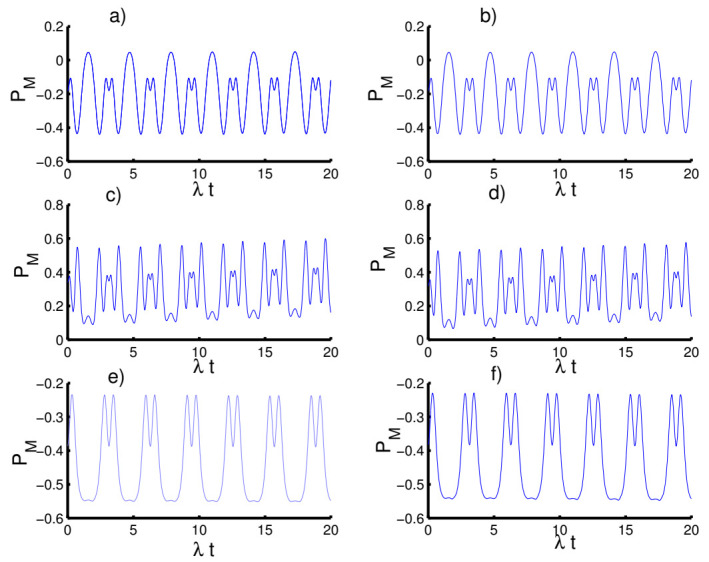
Effect of time-dependent coupling (p=1) on the evolution of the Mandel parameter $P_{M}$ for the physical parameters with the values (Z, χ)=(3,0). (**a**,**c**,**e**) The field initially in the CSs for PL potentials (h=0) and (**b**,**d**,**f**) show the field initially in the superposition of CSs for PL potentials (h=1). (**a**,**b**) Harmonic well potential (k=2, g=2)), (**c**,**d**) triangular well (k=1, g=3), and (**e**,**f**) infinite well (k→∞, g=4).

When the time dependence of the coupling is taken into account, as seen in Figure 8, the behavior of the Mandel parameter is periodic during the evolution, exhibiting sub-Poissonian and super-Poissonian distributions. Furthermore, the Mandel parameter behaves the same as before in all three cases.

**Figure 9:**
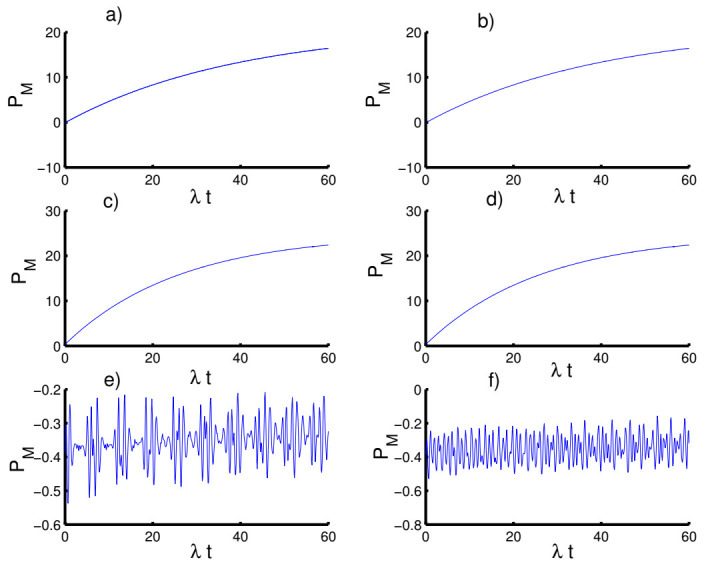
Effect of Kerr medium (χ=0.5) on the evolution of the Mandel parameter $P_{M}$ for the physical parameters with the values (Z, p)=(3,0). (**a**,**c**,**e**) The field initially in the CSs for PL potentials (h=0) and (**b**,**d**,**f**) show the field initially in the superposition of CSs for PL potentials (h=1). (**a**,**b**) Harmonic well potential (k=2, g=2), (**c**,**d**) triangular well (k=1, g=3), and (**e**,**f**) infinite well (k→∞, g=4).

The situation is quite different with respect to the influence of the Kerr medium on the Mandel parameter, as seen in Figure 9. In the first and second cases, the Mandel parameter initially starts with a Poisson distribution and is followed by a super-Poisson distribution for both cases of h=0 and h=1. Whereas, in the third case, the function $P_{M}$ exhibits fluctuations, showing sub-Poissonian distribution during the dynamics.

## 5. Conclusions

We have considered the interaction between light and matter in the context of the time-dependent interaction. We have presented a valuable and relevant new study of entanglement measures for a system of two qubits interacting with a field. We have used the Tavis-Cummings model considering two atoms when they simultaneously interact with a single-mode field in PL potentials and taking into account the effect of the time-varying coupling and Kerr medium. We have solved the Schrödinger equation to obtain the density operator that allows us to investigate the dynamical behavior of the quantumness measures such as von Neumann entropy, negativity and Mandel’s parameter. We have explained how the quantumness measures for the proposed scheme can be affected by the main parameters of the model, and we have compared the obtained results to the case of fields for different values of the exponent parameter of the PL potential. We have examined the time evolution of the atoms-field entanglement, atom-atom entanglement and the distribution of the photons in the field. We have found that the enhancement and preservation of the atoms-field entanglement and atom-atom entanglement can be achieved by a proper choice of the initial parameters of the field in the absence and presence of the time-dependent interaction and Kerr medium. On the other hand, we have determined the situations for which the field exhibits super-Poissonian, Poissonian or sub-Poissonian distribution. Moreover, we have displayed the dependence between the time variation of the entanglement and photons distribution according to the main parameters of the physical model for three values of the exponent parameter of the PL potentials. The proposed model can be useful to understand some quantum-mechanical phenomena of nonlinear optics. Finally, we consider only bipartite correlations, a study of multipartite-system correlations will be a useful contribution to understanding the dynamics of the information. An interesting contribution is to study the dynamic behavior of the quantumness measures for N-qubit system in interaction with fields in the framework of PL potentials. Another significant future investigation will be the study of the influence of finite-temperature environments on the dynamics of these measures.

## Data Availability

Not applicable.
